# Remarkably rapid, recent diversification of *Cochemiea* and *Mammillaria* in the Baja California, Mexico region

**DOI:** 10.1002/ajb2.16048

**Published:** 2022-09-04

**Authors:** Peter B. Breslin, Martin F. Wojciechowski, Lucas C. Majure

**Affiliations:** ^1^ Arizona State University School of Life Sciences 427 East Tyler Mall Tempe Arizona 85287 USA; ^2^ University of Florida Herbarium Florida Museum of Natural History 379 Dickinson Hall, 1659 Museum Rd. Gainesville Florida 32611 USA

**Keywords:** ancestral state reconstruction, Baja California, biogeography, Cactaceae, endemism, evolution, Mammilloid clade, rapid diversification, Sonoran Desert

## Abstract

**Premise:**

The Cactaceae of northwestern Mexico and the southwestern United States constitute a major component of the angiosperm biodiversity of the region. The Mammilloid clade, (Cactaceae, tribe Cacteae), composed of the genera *Cochemiea*, *Coryphantha*, *Cumarinia*, *Mammillaria*, and *Pelecyphora* is especially species rich. We sought to understand the timing, geographical and climate influences correlated with expansion of the Mammilloid clade, through the Sonoran Desert into Baja California.

**Methods:**

We reconstructed the historical biogeography of the Mammilloid clade, using Bayesian and maximum likelihood methods, based on a strongly supported molecular phylogeny. We also estimated divergence times, the timing of emergence of key characters, and diversification rates and rate shifts of the Mammilloid clade.

**Results:**

We found that the most recent common ancestor of *Cochemiea* arrived in the Cape region of Baja California from the Sonoran Desert region approximately 5 million years ago, coinciding with the timing of peninsular rifting from the mainland, suggesting dispersal and vicariance as causes of species richness and endemism. The diversification rate for *Cochemiea* is estimated to be approximately 12 times that of the mean background diversification rate for angiosperms. Divergence time estimation shows that many of the extant taxa in *Cochemiea* and Baja California *Mammillaria* emerged from common ancestors 1 million to 200,000 years ago, having a mid‐Pleistocene origin.

**Conclusions:**

*Cochemiea* and *Mammillaria* of the Baja California region are examples of recent, rapid diversification. Geological and climatic forces at multiple spatial and temporal scales are correlated with the western distributions of the Mammilloid clade.

The high absolute rates of diversification of angiosperms have been noted in several studies, especially since the advent of the use of molecular data to reconstruct evolutionary patterns through time (e.g., Crane et al., 1995; Davies et al., 2004; Soltis and Soltis, 2004; Magallón and Sanderson, 2007; Magallón and Castillo, 2009; Bell et al., 2010; Vamosi and Vamosi, 2011; Landis et al., 2018). Research continues regarding the mechanistic drivers of angiosperm speciation (such as vicariance caused by plate tectonics or orogeny, and climate cycles at multiple temporal scales) (e.g., Chaboureau et al., 2014), as well as biotic factors such as trait development, pollinator syndromes, and ploidy (e.g., van der Niet and Johnson, 2012; Serrano‐Serrano et al., 2017; Landis et al., 2018). The emerging field of geogenomics has the potential to provide strong inference regarding mechanistic forces that contribute to evolutionary histories and diversification rates (Dolby et al., 2022). Studies focused on Cactaceae present strong inference that the North American radiation of this angiosperm plant family has been particularly species‐rich, as well as characterized by recent dispersal and divergence times, particularly during the early‐to‐mid Pleistocene (e.g., Arakaki et al., 2011; Vázquez‐Sánchez et al., 2013; Hernández‐Hernández, 2014; Majure et al., 2019).

The historical biogeography of the flowering plant family Cactaceae Juss. Has been inferred from the current distributions of well‐supported clades, with estimates for crown and stem node ages derived from calibrations using taxa outside of the family, since there are no fossils of cacti (Wallace, 1995; Hershkovitz and Zimmer, 1997; Applequist and Wallace, 2001; Edwards et al., 2005; Ocampo and Columbus, 2010; Arakaki et al., 2011; Hernández‐Hernández et al., 2014; Barthlott et al., 2015). In spite of this prior research, a comprehensive family‐wide, well‐resolved phylogeny including all major clades and appropriate outgroups has not yet been constructed to test hypotheses of the early biogeography of Cactaceae. Similar to other families in the large, diverse order Caryophyllales in which it is nested, Cactaceae show multiple resilient adaptations to a wide variety of arid environments, including CAM photosynthesis, a waxy cuticle, and both chemical and morphological defenses against herbivory (Gibson, Noble, 1986; Mauseth, 1990; Anderson, 2001; Nobel, 2002; Soltis and Soltis, 2004; Mauseth, 2006; Smith et al., 2018). The dispersal into the Sierra Madre Oriental and the desert regions of north‐central Mexico coincides with increasing aridification and the decrease of atmospheric CO_2_ in the Late Miocene, approximately 12 Ma (million years ago) (Vázquez‐Sánchez et al., 2013). Aridification and reduced atmospheric CO_2_ correlate with rapid dispersal, increased diversification rates, and a high rate of speciation in the Cactaceae, presumably due to the competitive advantage provided by CAM photosynthesis and the development of highly efficient water storage tissue (Crozier, 2005; Arakaki et al., 2011; Vázquez‐Sánchez et al., 2013; Hernández‐Hernández et al., 2014).

Early estimates of the age of Cactaceae were made based on major geological events, such as the separation of Gondwana during the late Jurassic and early Cretaceous, approximately 145 Ma, because the family is entirely a New World lineage (with the exception of *Rhipsalis baccifera* (Sol.) Stearn, on the African continent, in Madagascar, and Sri Lanka, but dispersed from the Americas, cf. Cota‐Sánchez and Bomfim‐Patrício, 2010). Molecular dating of the Caryophyllales, in which Cactaceae are nested, indicates an origin of 116 to 104 Ma for that order (Anderson et al., 2005; Bell et al., 2010). More specifically, recent analysis of phylogenomic data from all 40 families within Caryophyllales indicates an origin of that order from 117 to 114.4 Ma (Magallón et al., 2015; Yao et al., 2019). The lack of a fossil record for Cactaceae introduces uncertainty into the time calibration of phylogenetic trees. However, Arakaki et al. (2011) used high‐confidence fossils from the Caryophyllales and other major clades to arrive at a well‐supported inference of a stem age of Cactaceae of about 35 Ma.

A recent phylogenetic reconstruction of the Mammilloid clade (Breslin et al., 2021) found that a clade of approximately 40 taxa formerly treated in the genus *Mammillaria* (sensu Haw., 1812) arose from a different most recent common ancestor than that of *Mammillaria* sensu stricto (s.s.). Those taxa were transferred to an expanded circumscription of the genus *Cochemiea* (K. Brandegee) Walton, hereafter referred to as *Cochemiea* (s.l.) (Figure 2, Clade “C,” in Breslin et al., 2021), as a result of that study. Most of these taxa occur on or are entirely endemic to the Baja California peninsula and adjacent islands in the Gulf of California and Pacific Ocean (Rebman and Roberts, 2012). In this study, the Mammilloid clade (Butterworth and Wallace, 2004) is well‐supported and composed of *Cochemiea* (K. Brandegee) Walton s.l., the Coryphantha clade, *Cumarinia* (F.M. Knuth) Buxb., and *Mammillaria* Haw. s.s. (Breslin et al., 2021). Note that we use the name Coryphantha clade in the broad sense to refer to the genus *Coryphantha* (Englem.) Lem. Plus taxa sometimes treated in the genus *Escobaria* Britton & Rose, now transferred to the genus *Pelecyphora* C. Ehrenb. (cf. Breslin et al., 2021; Sánchez et al., 2022), as well as the sister species to the rest of the clade, *Mammillaria sphacelata* Mart. (Figure 2, Clade “B”, in Breslin et al., 2021).

Arakaki et al. (2011) placed the core tribe Cacteae (in which the Mammilloid clade is nested) arising around 12 Ma, and the Mammilloid clade as emerging 7.6 to 6.3 Ma. Hernández‐Hernández et al. (2014) found similar crown ages for Cacteae, at approximately 12 Ma, and placed the origin of the Mammilloid clade at 8.6 to 7.3 Ma. Vázquez‐Sánchez et al. (2013) suggested an older origin for Cacteae, at 16 Ma, followed by increased diversification for the Mammilloid clade corresponding to increased aridity in the Late Miocene, approximately 6 Ma. Vázquez‐Sánchez et al. (2013) also found that the Baja Californian distribution of the former circumscription of the genus *Cochemiea* s.s. likely resulted from a jump dispersal event directly from the Mexican Plateau (Vázquez‐Sánchez et al., 2013).

The Mammilloid clade is sister to a clade in Cacteae containing the well‐supported, morphologically diverse and monotypic or often species‐poor genera *Acharagma* (N.P. Taylor) Glass, *Ariocarpus* Scheidw, *Chichimecactus* (Britton & Rose) Bárcenas, *Lophophora* J.M. Coult., *Obregonia* Fric., *Rapicactus* Buxb. & Oehme, *Strombocactus* Britton & Rose, and *Turbinicarpus* Buxb. & Backeb. (Butterworth et al., 2002; Butterworth and Wallace, 2004; Bárcenas et al., 2011; Hernández‐Hernández et al., 2011; Vázquez‐Sánchez et al., 2013; Bárcenas et al., 2021; Breslin et al., 2021). This sister clade to the Mammilloid clade is biogeographically restricted almost without exception to the Mexican Plateau, through the Sierra Madre Oriental, into the Chihuahuan and Coahuilan regions of Mexico and the United States (Anderson, 2001; Vázquez‐Sánchez et al., 2013).

## HABITATS AND CONTEMPORARY RANGE OF THE MAMMILLOID CLADE

In their current distributions, the taxa in the Mammilloid clade display adaptation to almost every available habitat from the equator northward, between the Pacific Coast of North America to roughly –97^○^ longitude west, south of latitude 43^○^ north. Four regions in North America have been identified as areas of high species richness in the Cactaceae—the Chihuahuan, Puebla‐Oaxacan, Jaliscan, and Sonoran‐Sinaloan regions of Mexico (Barthlott et al., 2015; Hernández and Gomez‐Hinostrosa, 2015). In the first three of those regions, the genera *Cochemiea* s.l., *Coryphantha*, and *Mammillaria* s.s. account for the greatest species diversity. The center of diversity for *Cochemiea* s.l. is on the Baja California peninsula itself, with approximately 28 out of the total of approximately 38 taxa in that genus endemic to the peninsula or its Gulf of California and Pacific Ocean islands along the west coast of Baja California (Rebman and Roberts, 2012; Barthlott et al., 2015; Hernández and Gomez‐Hinostrosa, 2015; Breslin et al., 2021). The Baja California peninsula, with a total area of about 144,000 km^2^, is noted for geographical and climatic heterogeneity over relatively small spatial scales, with as many as 11 distinct phytogeographic regions (Rebman and Roberts, 2012).

The primary goal of this study was to estimate the most likely history of the westward radiations of the Mammilloid clade, with a particular focus on the timing of the distributions of *Mammillaria* s.s. and *Cochemiea* s.l. into the Baja California region. We also sought to determine the historical biogeography of subclades in the Baja California region and estimate the relative impacts of dispersal and vicariance in contributing to species richness and endemism. To further illuminate the evolutionary history of the Mammilloid clade, we estimate divergence times, diversification rates, and rate shifts, and test whether or not key morphological characters are significantly correlated with diversification rate shifts.

## MATERIALS AND METHODS

### Taxon sampling

Our analyses included 89 samples, with 39 taxa from *Cochemiea*, and 16 taxa from *Mammillaria*, representing a regionally nearly complete taxon sampling from Baja California. A few infraspecific taxa from Baja California, as well as *Cochmiea mazatlanensis* (K. Schum.) D. Aquino & Dan.Sanchez, were unavailable for sampling. Representative samples of *Mammillaria* s.s. were selected from each of the major subclades recovered in the phylogeny of Butterworth and Wallace (2004). Fifteen members of the Coryphantha clade were sampled, including *Mammillaria sphacelata*, which has been recovered as sister to that clade (Breslin et al. 2021). Eight taxa from the genera *Acharagma*, *Ariocarpus*, *Lophophora*, *Rapicactus*, *Strombocactus*, and *Turbinicarpus* were selected as outgroups based on relationships shown in previously published phylogenies (Butterworth and Wallace, 2004; Barcenas et al., 2011; Vázquez‐Sánchez et al., 2013) and further verified by a molecular phylogeny of the cactus family based on analyses using the plastid *matK* gene (L. C. Majure, University of Florida, unpublished data). Taxon voucher information can be found in Breslin et al. (2021).

### DNA extractions, sequencing, assembly, and alignment

A modified CTAB procedure (Doyle and Doyle, 1987), with silica‐column based purification steps (Neubig et al., 2014; Majure et al., 2019) was used to extract total genomic DNA from silica dried plant material. Rapid Genomics (Rapid Genomics LLC, Gainesville, Florida, USA) performed library preparation and sequencing on the Illumina HiSeq X platform. Data acquisition was via a genome skimming process (Straub et al., 2012; Ripma et al., 2014; Majure et al., 2019). A majority consensus sequence was constructed from raw reads (150 bp, paired end) of *Mammillaria prolifera* (Mill.) Haw. assembled to the large single copy region (LSC) of a de novo assembly of the *Cylindropuntia bigelovii* (Engelm.) F.M. Knuth plastome (Majure et al., 2019), using Geneious Prime 2019.1.3 (Biomatters Ltd., Auckland, New Zealand). Manual adjustment of the alignment that resulted from initial automated alignment in MAFFT version 7 (Katoh and Kuma, 2002), was conducted in Geneious Prime 2019.1.3. Gaps were treated as missing data in all subsequent analyses.

### Phylogenetic analysis

Phylogenetic analyses were performed on our alignment of the large single copy region using RevBayes version 1.1.1 (Höhna et al., 2016). We had the benefit of a well‐supported tree topology derived from maximum likelihood, maximum parsimony, and Bayesian methods from our previous study (Breslin et al., 2021) for comparison. We implemented the GTR + Γ (gamma) nucleotide substitution model in RevBayes, based on results from jModelTest version 2.1.10 (Darriba et al., 2012). The RevBayes analysis used 183 different moves in a random move schedule with 240 moves per iteration, with 50,000 iterations in a single chain, for a total of 12 million moves. A maximum clade credibility tree with a 95% confidence interval of posterior probabilities was generated in RevBayes after discarding approximately the initial 25% of samples, based on visual inspection of log files in Tracer version 1.7.1 (Rambaut et al., 2018). The 95% clade credibility tree, as well as the set of 3750 post burn‐in trees, was used as the fixed topology to estimate divergence times, and the resulting time tree was used in subsequent biogeographic analyses. In analyses requiring an ultrametric binary branching tree, we used the divergence time tree, adjusted to resolve random polytomies in the R package ape 5.0 (Paradis and Schliep, 2019).

### Divergence times, ancestral character states, and diversification

We used two analyses to estimate divergence times. The first was performed in BEAST version 1.10.4 (Drummond and Rambaut, 2007; Drummond et al., 2012) and its associated software, BEAUti, Log Combiner, and Tree Annotator, with post‐run analysis to test for stationarity and time to burn‐in in Tracer version 1.7.1 (Rambaut et al., 2018). A test of the strict clocklike model was performed in PAUP* version 4.0a, build 165 (Swofford, 2002) using the likelihood ratio of the difference between clocklike and non‐clocklike evolution. The BEAST run included two taxon groups: (1) outgroups from the sister clade to the Mammilloid clade (including eight taxa from *Ariocarpus*, *Lophophora*, *Rapicactus*, *Strombocactus*, and *Turbinicarpus*); and (2) the Mammilloid clade, with 81 taxa. Tree priors were set to a mean of 12 Ma for outgroups and 7.5 Ma for the Mammilloid clade, based on Arakaki et al. (2011), with a normal distribution, following Drummond et al. (2012). Based on the best AIC and BIC scores in jModelTest version 2.1.10 (Guindon and Gascuel, 2003; Darriba et al., 2012), the GTR + Γ model for nucleotide substitution was selected, with the three codon positions unlinked. The clock model used was an uncorrelated relaxed clock (Drummond et al., 2006) with a lognormal distribution. Following Drummond et al. (2012), birth‐death and Yule pure birth analyses were compared via marginal log likelihood scores, to test which tree prior was more informative. The BEAST run included 20 million iterations with trees sampled every 2000 generations, from which, excluding trees from the burn‐in, as determined by Tracer, a 95% maximum clade credibility tree was constructed from 4280 trees.

To double check the divergence times estimated in the BEAST analysis, a second divergence time estimation was conducted in RevBayes, using an uncorrelated log normal model, a fixed topology from the 95% clade credibility tree derived from the RevBayes GTR + Γ analysis as the starting tree, and the root time set to 12 Ma, with the origin of the Mammilloid clade set to offset the root time by 7.5 million years, based on previous estimates from Arakaki et al. (2011) and Hernández‐Hernández et al. (2014). The RevBayes analysis ran for 25,000 iterations with a random move schedule of 190 moves and a total of 420 moves per iteration, for a total of 10.5 million moves. A majority rule consensus tree trace was generated in RevBayes.

To investigate whether the origin of morphological characters was correlated with lineage‐specific diversification rates, a binary state speciation and extinction (BiSSE) analysis and a hidden state speciation and extinction model (HiSSE) were analyzed in RevBayes (Maddison et al., 2007; Magnuson‐Ford and Otto, 2012; Höhna et al., 2019). The characters used in this analysis were chosen based on results from an earlier analysis in Mesquite version 3.6 (Maddison and Maddison, 2019), which showed strong genus‐level correlation of three characters for *Mammillaria*, *Coryphantha*, and *Cochemiea* (Breslin et al., 2021), respectively: (1) lactiferous ducts in the parenchyma of *Mammillaria*; (2) adaxial grooves on the tubercles of *Coryphantha*; and (3) hooked spines in *Cochemiea*. We used a log uniform distribution for diversification rates, and a flat Dirichlet prior on the root state, following Höhna et al. (2019). The analysis, adjusted for incomplete taxon sampling, ran for 50,000 iterations on two chains, with 20 moves per iteration, for each character state, across our entire tree. Post‐processing and analysis of the BiSSE and HiSSE results, including plotting posterior distributions of speciation rates and ancestral states of characters, was conducted using RevGadgets version 1.0.0 (Tribble et al., 2021). The HiSSE model was implemented in RevBayes to check for possible spurious correlation of the trait of hooked spines in *Cochemiea* s.l. with increased diversification rates (Beaulieu and O'Meara, 2016; Höhna and Freyman, 2022). The parameters of the HiSSE model were identical to those of the BiSSE model, with the addition of four hidden states.

A reversible jump Markov chain k‐character analysis (Mk) for the above‐mentioned characters (lactiferous ducts in *Mammillaria*, adaxial grooves in *Coryphantha*, and hooked spines in *Cochemiea* s.l.) was carried out in RevBayes to trace the most likely timing of their origin and their ancestral states through time. The analysis used the fixed tree used for all downstream analysis, with the rate matrix set for independent, exponentially distributed rates of character evolution, and a Dirichlet prior on the root state, following Höhna et al. (2022).

Diversification rates and rate shifts over time were estimated using both maximum likelihood and Bayes factors methods, in turboMEDUSA version 0.953 (Alfaro et al., 2009; Pennell et al., 2014), implemented in R version 4.0.5 (R Core Team, 2021) and BAMM 2.5.0 (Rabosky, 2014). In the TurboMEDUSA analysis, adjustment for incomplete taxon sampling in outgroups, *Mammillaria*, and the Coryphantha clade was incorporated into model runs. In addition to our sampled taxa, the number of currently accepted species in each clade or genus was used to approximate clade richness (outgroups, 30; *Mammillaria*, 124; the Coryphantha clade, 55) (Dicht and Lüthy, 2005; Hunt, 2016; Vázquez‐Sánchez et al., 2019; Breslin et al., 2021). Automated optimal model selection, forced birth‐death and pure birth (Yule) models were generated for comparison. For the BAMM analysis, priors were set using the R package BAMMtools version 2.1.8 (Rabosky et al., 2014), and the analysis ran four chains with 1 × 10^9^ iterations, writing states every 400,000 iterations. Convergence was evaluated visually using the R package coda version 0.19‐4 by inspecting a plot of log likelihoods for each generation, after discarding a burn‐in of the first 10% of states. A total of 2250 post burn‐in states and an effective sample size of 451 samples were used for analysis. We used an incomplete sampling scheme to reflect clade species richness, assigning a sampling probability of 23% for outgroups, 15% for *Mammillaria*, 20% for the Coryphantha clade, 100% for *Cumarinia*, and 90% for *Cochemiea*, with each probability derived from the ratio of the number of samples in our tree to the number of taxa in each clade. Downstream analysis included inspection of Bayes factors for multiple models, plotting the maximum a posteriori probability shift tree, and calculating clade‐specific diversification rates and 95% confidence intervals.

### Reconstructing ancestral biogeography

Methods used for probabilistic historical biogeographical reconstruction included Bayesian inference for discrete areas (BayAreas; Landis et al., 2013), statistical dispersal‐vicariance analysis (S‐DIVA; Yu et al., 2010), statistical dispersal‐extinction‐cladogenesis (S‐DEC) (Ree et al., 2005; Ree and Smith, 2008), and analysis of biogeography in RevBayes, approximately following Landis et al. (2018). Each of these methods uses either maximum likelihood or Bayesian posterior estimates of the ancestral geographic ranges of internal nodes on a time calibrated phylogenetic tree, based on stepwise hindcasting from current ranges of the taxa at the tree tips (cf. Sanmartín et al., 2008). S‐DIVA and S‐DEC utilize a multiple tree sampling method, for which we used 3000 dichotomous trees randomly selected from 3750 post burn‐in trees from our RevBayes analysis described above. Other methods used the 95% clade credibility tree from our RevBayes analysis. As we had an identical tree topology derived from multiple previous methods (Breslin et al., 2021), and the RevBayes analysis was 100% congruent with those topologies, we used a fixed tree topology for all analyses. All biogeographical analyses except the RevBayes analysis were carried out in RASP version 4.2 (Yu et al., 2020). The clearest and most detailed reconstructions of historical biogeography used nine ancestral ranges, based on current distributions of the taxa sampled here, and delimited according to World Wildlife Fund level III ecoregions (Olson et al., 2001). The ranges used were: (1) eastern Mexico (a composite of six level III ecoregions, with major regions being the Mexican Plateau, the Sierra Madre Orientale, and the Coahuilan and Chihuahuan Deserts); (2) the Sonoran Desert region; (3) the Mojave Desert region; (4) the Sonoran‐Sinaloan coastal scrub region; (5) the California Floristic Province; (6) the Gulf Coast and Central Desert of Baja California (including the islands in the Gulf of California); (7) the Pacific Coast and Pacific islands of Baja California; (8) the Cape Region of Baja California; and (9) the Trans‐Mexican Volcanic Belt and all areas south of that region. These ranges span five adjacent non‐peninsular major floristic domains of the Mammilloid clade and outgroups (Vázquez‐Sánchez et al., 2013), and four sharply delineated Baja California phytogeographic regions, including regions recognized as biodiversity and endemism hotspots (Riemann and Ezcurra, 2007; Rebman and Roberts, 2012).

In all methods, connectivity (that is, the ability of taxa to occupy or disperse among ancestral ranges) was limited to a maximum of three areas, to approximate the currently known maximum dispersal distance of Mammilloid taxa based on current range size. Results from the S‐DIVA, S‐DEC, and BayAreas analyses were summarized by using the most likely ancestral ranges for each node. For the RevBayes analysis, the top three most likely ancestral ranges were considered, based on Bayesian posterior probabilities.

Graphical representation of the most likely ancestral range was implemented by color coding node pies to correspond with colored ancestral ranges on a map of our study area. In the case of composite ranges, a single geographical area was chosen for the node pie based on comparison across all four methods, when possible. Nodes with roughly equal probabilities for multiple ranges across methods, or high uncertainty of the ancestral range, with total estimates below 40%, were left uncolored, but with probabilities of ranges indicated.

## RESULTS

### Alignment and tree topology

The alignment of the large single copy plastid sequences used in all analyses contained a total of 93,808 characters, with 22,218 of these characters variable, of which 9287 characters were uninformative, and 12,931 were parsimony informative.

The topology of all trees generated in analyses described here were congruent with the topology derived from previous maximum likelihood, maximum parsimony, and Bayesian analyses of the Mammilloid clade (Breslin et al., 2021). All nodes in the 95% clade credibility trees generated in RevBayes or BEAST had a Bayesian posterior probability of 0.95 or higher. The 50% majority consensus tree generated for S‐DEC and S‐DIVA analyses in RASP had bootstrap support values of 80% or higher for all nodes (Figure 2).

### Divergence time estimates

**Figure 1:**
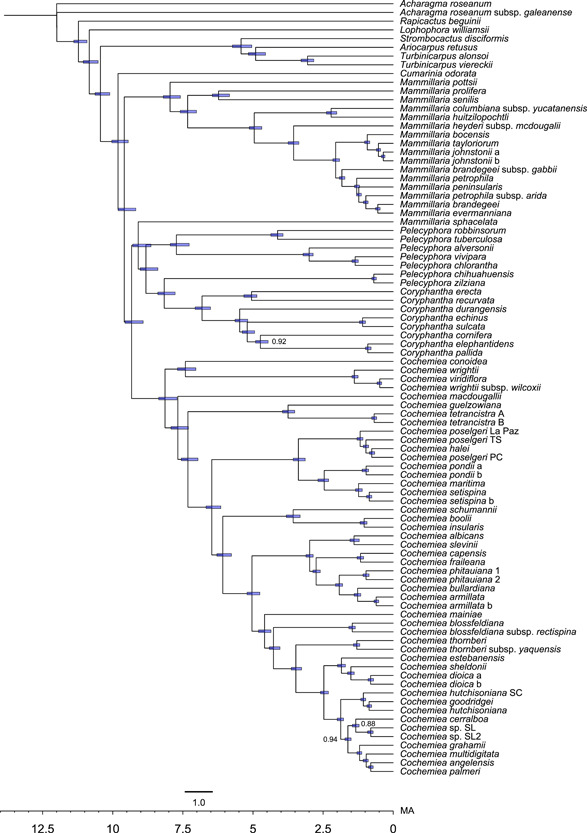
Divergence time estimates for the Mammilloid clade with 95% confidence intervals. The tree shown resulted from the divergence time analysis in RevBayes using a fixed tree topology, deploying the GTR + Γ (gamma) tree resulting from Bayesian analysis in RevBayes. Node bars show 95% confidence intervals for divergence times. The time scale at the bottom of the tree is in millions of years, calibrated to a root time of 12.5 Ma, offset by 7 myr for the origin of the Mammilloid clade. Posterior probabilities of clade support are 1.0 unless indicated.

In both the RevBayes and BEAST analyses, the 95% confidence interval provides an estimate for the divergence of the Mammilloid clade crown group (including *Cumarinia*) at 7.5 to 8 Ma. The subsequent divergence of the Coryphantha clade, *Mammillaria*, and *Cochemiea* was estimated to have occurred roughly simultaneously, at approximately 7 to 7.5 Ma. The crown age for *Cochemiea* was approximately 5.5 Ma, and *Mammillaria* diverged more recently at 4.5 Ma. The extant Baja California regional taxa in *Cochemiea* and *Mammillaria* are estimated to have speciated relatively recently, with 28 of 35 taxa arising between 1.0 Ma and approximately 200,000 years ago (Figure 1).

### Ancestral biogeography

**Figure 2:**
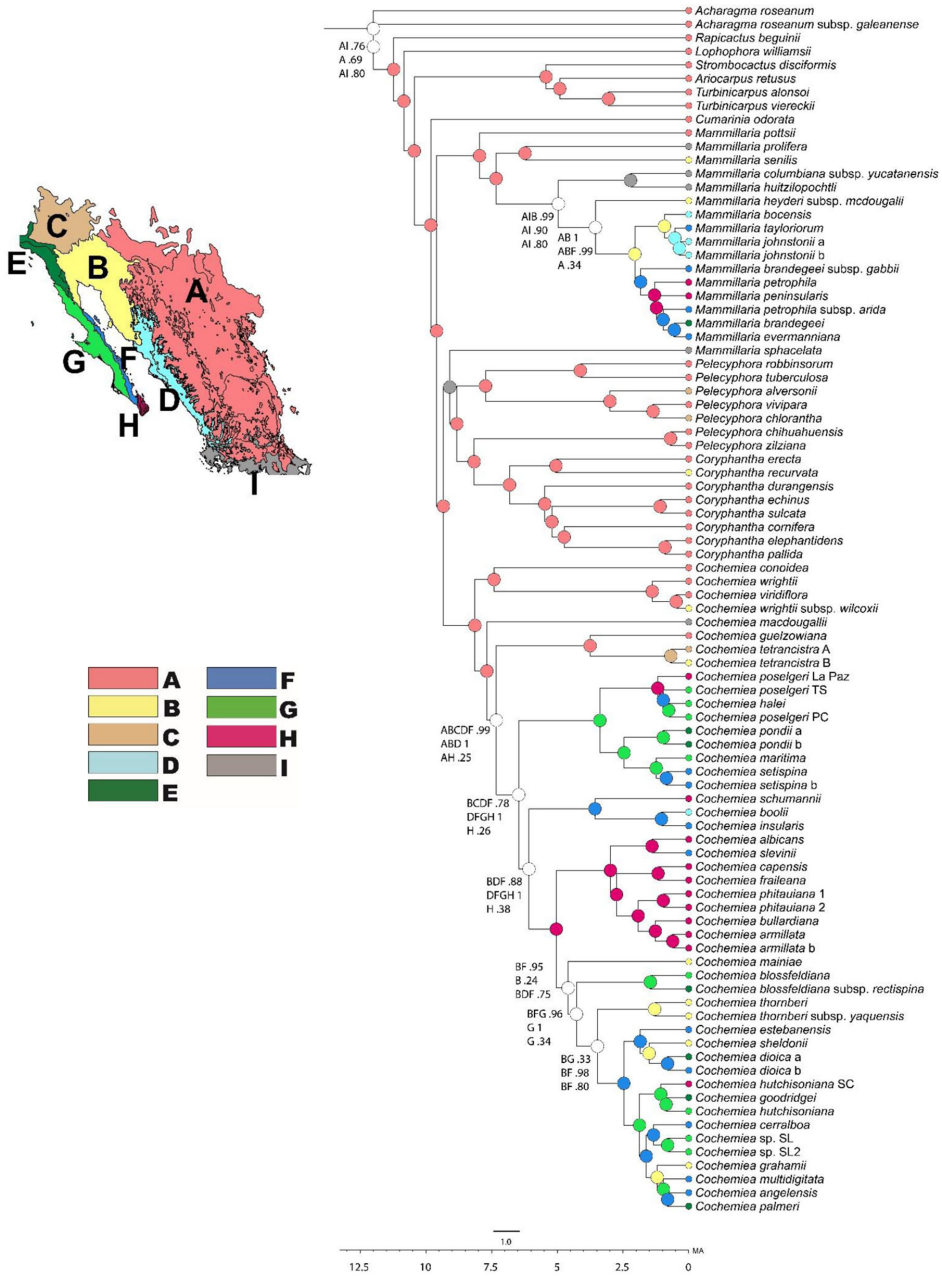
Ancestral biogeography of the Mammilloid clade. Ancestral ranges are color coded according to the map colors. Ranges are: A: Eastern Mexico; B: Sonoran Desert; C: Mojave Desert, D: Sonoran‐Sinaloan Coastal Thornscrub; E: California Floristic Province; F: Gulf of California Islands and Coastal Desert; G: Pacific Coast; H: Cape Region of Baja California; and I: South of the Trans‐Mexican Volcanic Belt. Solid colored nodes indicate 80% or higher support for that ancestral range across all methods. Uncolored nodes represent multiple ranges and/or disagreement among methods. Ranges for these nodes are given according to S‐DEC, S‐DIVA, and RevBayes analyses, respectively. The time scale at the base of the tree is in millions of years. The tree is the time tree generated in RevBayes as shown in Figure 1.

The most recent common ancestor (MRCA) of the Mammilloid clade originated in Eastern Mexico (region A), approximately 8 to 7.5 Ma. The most recent common ancestor of *Mammillaria* s.s. occurred in eastern Mexico as well, at 7.5 to 7 Ma. The most recent common ancestor of the Coryphantha clade originated either in eastern Mexico or a composite range including eastern Mexico and south of the Trans‐Mexican Volcanic Belt (regions A and I), approximately 7.5 Ma. The MRCA of *Cochemiea* s.l. also originated in eastern Mexico, 7 to 6 Ma. The timing of differentiation among *Mammillaria* s.s., the Coryphantha clade, and *Cochemiea* s.l. began within the 95% confidence intervals of divergence times for their most recent common ancestors, so could have been nearly simultaneous (Figures 1 and 2).

The Mammilloid clade did not begin to disperse out of eastern Mexico until approximately 6 Ma. The first extant genus from the Mammilloid clade to have become distributed in the Sonoran Desert (region B) was *Mammillaria* s.s., with the most recent common ancestor of a subclade containing only those *Mammillaria* currently distributed in Baja California and the Sonoran Desert at about 5 to 4.5 Ma (Figure 2, Baja Mammillaria Clade). *Mammillaria* s.s. first occurred in an exclusively Baja California peninsular range, specifically in the Baja California Gulf Coast (region F) and the Baja California Pacific Coast (region G), between 3.5 to 3 Ma. From approximately 3 Ma to the present, *Mammillaria* s.s. has undergone multiple dispersal events to the Gulf Coast and Cape Regions of Baja California, as well as to the Sonoran Desert and Sinaloan Coastal regions. The *Mammillaria* s.s. taxon with the most northwestern Baja Californian distribution, *Mammillaria brandegeei* Engelm. ex K. Brandegee, arrived in its current distribution via the Gulf Coast of Baja California, perhaps as recently as 1 Ma (Figure 2).

The Coryphantha clade has largely remained in eastern Mexico (region A), with a biogeography similar to the sister clade to the Mammilloid clade. All taxa in the Coryphantha clade with current distributions in the Sonoran (region B) and the Mojave Deserts (region C) have most recent common ancestors occurring in eastern Mexico (region A). Distribution of members of the Coryphantha clade outside of eastern Mexico occurred approximately 3 to 2 Ma (Figure 2; Coryphantha clade). In our phylogenetic reconstruction, *Pelecyphora* in the sense of Sanchez et al. (2022); that is, including *Escobaria*, is paraphyletic, perhaps a result of our low sampling coverage of that clade. The taxon *Escobaria chlorantha* (Engelm. ex Wheeler) Buxb., nested in *Pelecyphora*, treated by Sanchez et al. (2022) as a synonym of *Pelecyphora vivipara* Sanchez, we treat at species rank, and combine here as *Pelecyphora chlorantha* (Engelm. ex Wheeler) P.B. Breslin & Majure, comb. nov.

*Cochemiea* s.l. began dispersal to the west from its eastern Mexican origins approximately 5.5 Ma. The subclade containing *C. conoidea* (DC.) P.B. Breslin & Majure, *C. wrightii* (Engelm.) Doweld, *C. wrightii* subsp. *wilcoxii* (K. Schum.) Doweld, *C. viridiflora* (Britton & Rose) P.B. Breslin & Majure, *C. macdougallii* (Alexander) P.B. Breslin & Majure, *C. guelzowianai* (Werderm.) P.B. Breslin & Majure, and *C. tetrancistra* (Engelm.) P.B. Breslin & Majure, however, is distinguished by remaining largely in eastern Mexico (region A), or having limited distributions in the Sonoran (region B) or Mojave deserts (region C) (Figure 2; Conoidea Clade).

In contrast, the most recent common ancestor for taxa in *Cochemiea* s.l. currently distributed from eastern Mexico to the west, in the Sonoran Desert (region B) or the Baja California regions (regions E–H) have a most recent common ancestor with a likely range outside of eastern Mexico about 500,000 years more recently, at about 5 Ma. Between approximately 5 Ma and 3 Ma, the most recent common ancestor of a subclade containing *Cochemiea* s.s. (i.e., *C. halei* (K. Brandegee) Walton, *C. maritima* H.E. Gates ex Shurly, *C. pondii* Walton, *C. poselgeri* (Hildm.) Britton & Rose, and *C. setispina* Walton) arrived on the Baja California peninsula. This subclade is most likely to have arrived on the Pacific Coast of Baja California at 5 to 4 Ma (Figure 2; Cochemiea s.s. clade). The other earliest most recent common ancestor to have a Baja California range occurred in the Cape Region (region H) 3.5 to 3 Ma, the ancestor of a species‐rich subclade containing six taxa still endemic to the Cape Region (Figure 2; Cape clade), as well as 16 other Baja California taxa. The subclade containing *C. boolii* (G.E. Linds.) P.B. Breslin & Majure, *C. insularis* (H.E. Gates) P.B. Breslin & Majure, and *C. schumannii* (Hildm.) P.B. Breslin & Majure also had a most recent common ancestor on the Gulf Coast and the Cape Region of the Baja peninsula, dated at approximately 3 Ma (Figure 2; Schumannii Clade). The subclade within *Cochemiea* s.l. that comprise 16 taxa from *C. mainiae* (K. Brandegee) P.B. Breslin & Majure, through *C. angelensis* (R.T. Craig) P.B. Breslin & Majure, has a complex geographical history involving the Sonoran Desert region, beginning about 3 Ma, with the first fully peninsular ranges occurring about 1 Ma, either on the Gulf Coast or the Pacific Coast of Baja California (Figure 2; Mainiae clade). This clade also shows a history of ancestors on the peninsula dispersing again to the mainland, and is the most recently diversifying clade, with speciation indicated as recently as 200,000 years ago (Figure 2).

### BiSSE analysis of characters and diversification

No statistically significant correlation was found between the characters of lactiferous parenchymal ducts in *Mammillaria* s.s., or adaxial grooves in the Coryphantha clade, and changes in diversification rates in the BiSSE analyses. Analysis of character‐dependent changes in diversification using the binary state speciation and extinction (BiSSE) model suggested that hooked spines in *Cochemiea* s.l. were correlated with almost double the rate of diversification in that clade. Follow‐up analysis using the hidden state speciation and extinction model (HiSSE), however, showed weak correlation between the character of hooked spines and increased diversification (maximum pp < 20% of an approximate 20% increase in diversification rate).

**Figure 3:**
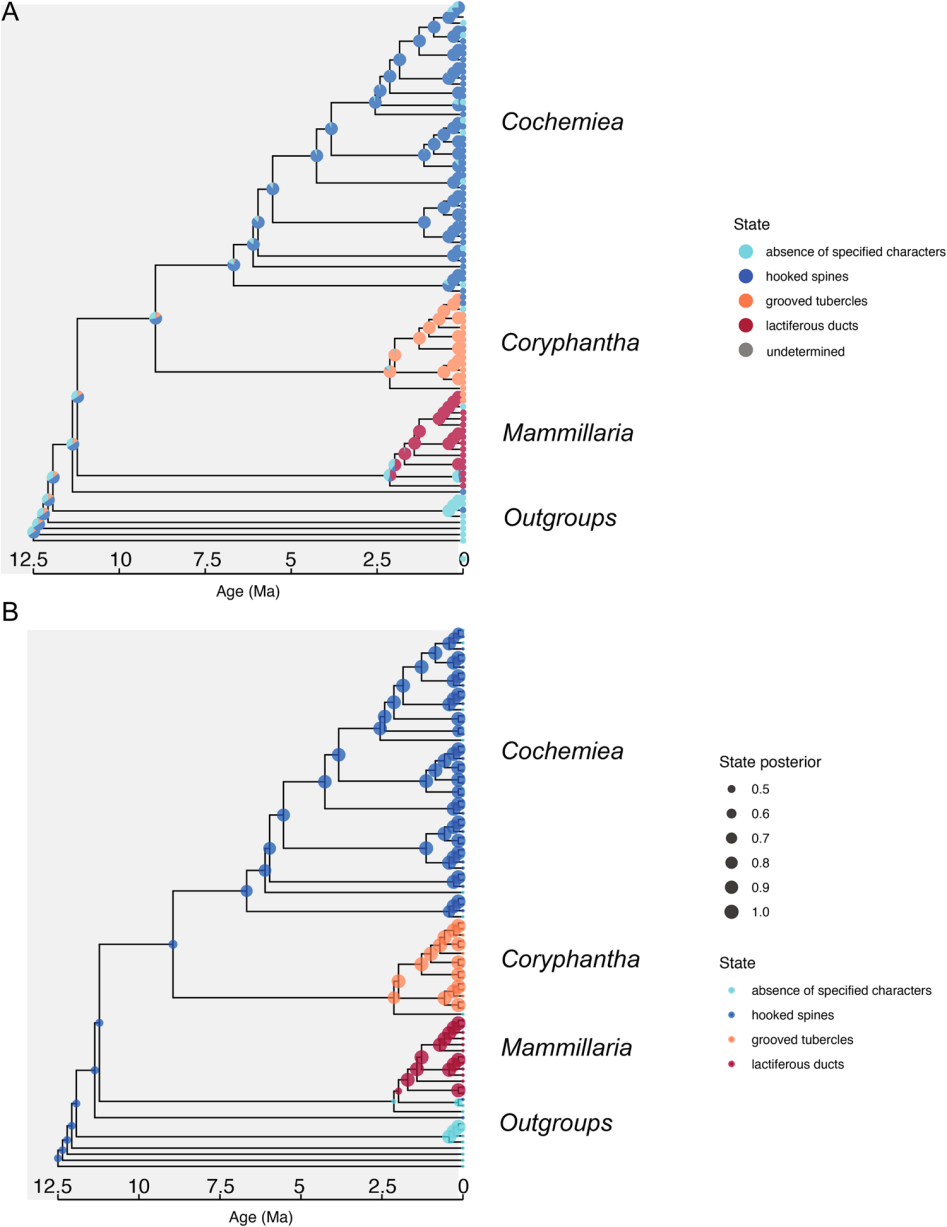
Ancestral state estimation for three characters in the Mammilloid clade. (A) all possible states at each node, color‐coded according to the legend, with node pie slice size equal to posterior probability; (B) the most likely state at each node based on posterior probability, scaled by node size. The results are from a reversible jump Markov chain k‐states analysis in RevBayes, with two chains, and 250,000 iterations, with 50 moves per iteration, for a total of 12.5 million moves, with posterior probabilities of character states estimated after a burn‐in of 10% of states. The clade labeled *Coryphantha* includes *Pelecyphora*.

The Mk ancestral states reconstruction showed that the distinguishing characters for the clades in the analysis originated at or soon after the most recent common ancestor for each clade, and have been highly consistent through time in each case, with posterior probabilities >0.90 at all nodes after the emergence of the trait (Figure 3). The Mk results revealed that it is most likely that existing members in *Cochemiea* s.l. that do not have hooked spines, and *Mammillaria senilis* Lodd. ex Salm Dyck of *Mammillaria* s.s., which has hooked spines have undergone those character changes very recently (Figure 3) and represent homoplasy.

### Diversification rates and rate shifts

Both the TurboMEDUSA and BAMM diversification models reported significant heterogeneity of diversification rates in the Mammilloid clade. The TurboMEDUSA analysis showed six likely significant diversification rate shifts and high absolute diversification for the Mammilloid clade, shortly after that taxon diverged at approximately 7 Ma, with a median diversification rate of 1.35 and a 95% confidence interval between 0.912 and 1.99. Conversely, the subclade of the Coryphantha clade (i.e., *Coryphantha* s.s.), separate from *Pelecyphora* (sensu Sánchez et al. 2022), is likely to have undergone a significant slowdown in diversification approximately 2.5 Ma, with a median diversification rate of 0.0005, with a 95% confidence interval between 0.0003 and 0.0012, while *Pelecyphora* s.l. maintained the diversification rate of the Mammilloid clade. This slowdown is in sharp contrast to the approximately simultaneous increase in diversification of the Baja California *Mammillaria*, with a rate shift for that subclade resulting in a median diversification rate of 2.75, with a 95% confidence interval between 2.11 and 3.65 (Table 1). In general, pure birth models were selected for optimal fit, and extinction rates were not estimated to have had a major influence on diversification for any of the clades or genera other than the core Cacteae as a whole and *Coryphantha* (Table 1). The BAMM analysis was largely consistent with the TurboMEDUSA results, showing high absolute and relative diversification rates for *Cochemiea* s.l. and the western distribution of *Mammillaria* s.s. The maximum a posteriori credible rate shift model indicated four rate shifts with relatively high marginal probabilities at approximately the same nodes, as indicated in four of the rate shifts in the TurboMEDUSA analysis. The credible number of rate shifts of all models ranged from 0 to 11. The overall mean rate of diversification for the Mammilloid clade was 0.962, with the 95% confidence interval between 0.693 and 1.290, and the clade‐specific mean diversification rate for *Cochemiea* s.l. was 1.15 with a 95% confidence interval between 0.895 and 1.454 (Table 1). The mean rate for the Baja Caifornia taxa in *Mammillaria* s.s. was more conservative than the TurboMEDUSA analysis at 1.06 with a 95% confidence interval between 0.805 and 1.34 (Table 1). The overall mean diversification rate for the outgroups was 0.553 with a 95% confidence interval between 0.096 and 1.24, a comparatively large range due to proposed increased diversification in *Turbinicarpus* and *Rapicactus* beginning approximately 1.5 Ma. The most significant difference between the TurboMEDUSA analysis and the BAMM analysis was that BAMM did not find support for the dramatic slowdown in diversification of *Coryphantha*.

**Table 1:** Diversification rates for the Mammilloid clade at most likely rate shifts

Shift	Clade	Age, Ma	TurboMedusa, Median r	ε	BAMM‐Mean r	BAMM 95% CI
	Angiosperms	160	(0.08)	0	(0.08)	na
1	Outgroups	13–1	1.54	0.788	0.553	0.096–1.24
2	Mammilloid clade	7.5	1.35	0	0.962	0.693–1.290
3	*Cochemiea*	7–5	1.99	0	1.15	0.895–1.454
4	Baja *Mammillaria*	3.5–2	2.75	0	1.06	0.805–1.34
5	*Coryphantha* *	7–5	0.0005	0.99	0.008	0.0062–0.0015

Notes: r = lineages per million years. ε = extinction rate/speciation rate. In cases where ε is 0 TurboMEDUSA's optimal model fit was a pure birth, a.k.a. Yule, model. The angiosperm diversification rate is the high estimate from Magallón and Sanderson (2007).

* The *Coryphantha* results are for the subclade of Coryphantha separate from *Pelecyphora*.

Relative diversification rates for *Cochemiea* s.l., using the more conservative estimates in BAMM, were at least twice as high as outgroups, and 1.2 times the Mammilloid clade as a whole. The Baja California *Mammillaria* were 1.92 times higher than outgroups, and 1.10 times higher than the Mammilloid clade as a whole. The Mammilloid clade in general had a mean diversification rate approximately 12 times higher than other estimates of angiosperm diversification (Table 1).

## DISCUSSION

The recent, rapid diversification and complex biogeographical history of one of the most species‐rich clades in the Cactaceae has multiple implications. Results presented here confirm earlier results regarding the divergence times of the Mammilloid clade by Arakaki et al. (2011) and Hernández‐Hernández et al. (2014), as well as recovering a history that provides time‐scaled and biogeographical insight into the molecular phylogeny of Breslin et al. (2021). Whereas *Mammillaria* s.s. and the Coryphantha clade have largely remained restricted to their ancestral ranges in eastern Mexico (region A), the dispersal across the Sonoran Desert region to the Cape Region, Gulf Coast, and Pacific Coast of Baja California resulted in *Cochemiea* s.l. evolving into a lineage distinct from *Mammillaria* s.s., with a unique Sonoran/Sinaloan and Baja California distribution, high species diversity and 90% peninsular endemism. This high diversity and endemism in Baja California coincides with multiple short‐ and mid‐range dispersal events into markedly different habitats entirely contained on the peninsula or adjacent Sonora/Sinaloa regions, characterized by unique topography and climate.

### Spatial and temporal scales of geological, geographical and climate forces

The geo‐tectonic processes that caused the separation of the Baja California peninsula from mainland Mexico by rifting are estimated to have begun more than 30 million years ago and are still ongoing (Dolby et al., 2015). Hypothetical mid‐peninsular seaways, perhaps indicated by north‐south genetic discontinuities, may have formed between 1 and 2 Ma (Dolby et al., 2015; Helenes, Carreño, 1999 and the references therein). More recent glacial‐interglacial cycles influencing gene flow and/or dispersal find biological support from current high genetic diversity in predicted glacial refugia, high recent gene flow among islands connected to the peninsula at the lowest sea levels, and postglacial range expansions (Dolby et al., 2015). Multiple geological forces over several time scales have contributed to the accelerated rate of plant diversity and endemism, with 30% of plant taxa endemic to the peninsula (Rebman and Roberts, 2012; Dolby et al., 2015). The peninsular endemism observed in the Cactaceae in general, and *Mammillaria* s.s. and *Cochemiea* s.l. in particular, is high, with 93 out of 130 taxa (species and subspecies), or 72%, endemic to the peninsula. *Cochemiea* s.l. with approximately 26 taxa endemic to Baja California or its associated Gulf of California or Pacific Islands, is the most species‐rich genus on the peninsula, with an endemism rate of 94%.

In this analysis, three factors support the strong effects of peninsular rifting on the evolution of *Cochemiea* s.l. Given that the highest probability for the original ancestral range for *Cochemiea* after its older eastern Mexican origins is the Cape Region, which is estimated to have been already approximately 200 km distant from the mainland at about 7 to 5 Ma (Bennett, 2013; Dolby et al., 2015), all of the analyses indicate correlation with this separation. A shift toward higher diversification rates at approximately 7 Ma is predicted by the BiSSE analysis, and strongly correlated with the development of hooked spines. Since *Cochemiea* emerged in eastern Mexico, results of the BiSSE and TurboMEDUSA analyses suggest that increased diversification rates for *Cochemiea* were in effect prior to western dispersal (Table 1).

On a more recent time scale, within *Cochemiea* s.l., there is little support for distinct north‐south genetic separation created by mid‐peninsular seaways at the species level, as all of the clades show equally likely distributions among taxa located either in the northern or southern portions of the peninsula. In fact, more recent nodes dated between 1 to 2 Ma show multiple north‐south and south‐north ancestral jumps, suggesting that, if a mid‐peninsular seaway formed 1 to 2 Ma, it had little effect on gene flow or dispersal. This is consistent with several mid‐range jump dispersals suggested by the ancestral range analysis, where larger geographical barriers than a mid‐peninsular seaway seem to have not been a hindrance (for example, the Gulf of California, within more recent times, when it has been at its widest, cf. Dolby et al., 2015).

The most likely ancestral range for the clade containing *Cochemiea* s.s. (i.e., the five taxa in *Cochemiea* sensu (K. Brandegee) Walton) is the Cape Region of Baja California, with a jump to the Pacific Coast of the Baja California peninsula at about 3 Ma, and then dispersal back to the Cape Region for *C. poselgeri* at 2.5 Ma, with the island dispersal of *C. halei* occurring as recently as 500,000 years ago. In spite of its recent dispersal to the archipelago in Bahía Magdalena, *C. halei* shows a suite of pronounced morphological differences from its sister taxon, *C. poselgeri*, as well as a facultative relationship with serpentine, a form of ultramafic ocean crustal rock that forms the bedrock of the island (Breslin et al., 2020 and the references therein). This suggests rapid adaptation to an environment markedly different from both the northern Pacific coast and the Cape Region of Baja California. We were unable to include the recently described and likely member of *Cochemiea* s.s., *Cochemiea thomasii* García‐Mor., Rodr. González, J. García‐Jim. & Iamonico (Garcia Morales et al., 2020), which, like *C. halei*, also has straight spines, and occurs approximately 250 km from the Cape Region of Baja California, in the Mexican state of Sinaloa (Garcia Morales et al., 2020). It remains to be seen whether *C. thomasii* represents a recent dispersal to mainland Mexico from the peninsula, or is perhaps ancestral to *Cochemiea* s.s.

An exception to multiple north‐south dispersals is exhibited by the clade composed entirely of Cape Region endemics (Figure 2; Cape clade). One of the putative trans‐peninsular seaways is an inundation that isolated the Cape Region as an island for approximately 1 million years, about 2 million years ago. This seaway has found some support from population level phylogenetic studies of lizards and other vertebrates (Upton and Murphy, 1997; Riddle et al., 2000). This hypothesis is supported here by the total endemism of the Cape clade and the timing of its divergence. It may also be that climate plays a crucial role in this endemism, however, since the tropical regime of the Cape is significantly distinct from adjacent regions. Further research into habitat suitability for the taxa of the Cape Region, as well as population level genetic analysis, would provide more insight into the total endemism of this clade.

Glacial/interglacial sea level changes more recently could be reflected in multiple east‐west dispersals, where lower sea levels would have facilitated trans‐Gulf dispersal or north‐south dispersal along the Pacific coast. For example, the most recently diverging taxa in *Cochemiea* are close relatives to the mainland‐only taxa *Cochemiea grahamii* (Engelm.) Doweld, *C. multidigitata* (Radley & G.E. Linds.) P.B. Breslin & Majure, *C. angelensis*, and *C. palmeri* (J.M. Coult.) P.B. Breslin & Majure. Each of these taxa is restricted, widely dispersed island endemics—*C. multidigitata* on San Pedro Nolasco Island, 16 km from the Sonoran coast, *C. angelensis* on Isla Angel de la Guarda, in the northern Gulf, and *C. palmeri* on the San Benitos Islands, in the Pacific, near Isla Cedros. Their estimated divergence is within the past 0.5 to 0.2 million years, and their east‐west island and cross‐peninsular dispersal may reflect dispersal or pollinator routes available at low sea levels, followed by isolation due to interglacial high sea levels. Within approximately 100,000 years, the most likely ancestral range of *C. angelensis* and *C. palmeri* is the northern Pacific coast of Baja. Several other island endemics—*C. goodridgei* (Scheer ex Salm‐Dyck) P.B. Breslin & Majure, *C. blossfeldiana* subsp*. rectispina* (E.Y. Dawson) P.B. Breslin & Majure, an unnamed taxon on Isla Magdalena, and *C. halei* also on Isla Magdalena—show divergence times in the BEAST analysis approximately coinciding with low sea levels during glacial periods. All of the estimates of dispersal events at less than approximately 500,000 years ago are within the margin of error of both the BEAST and RevBayes analyses, however, so they are a matter of conjecture. A more detailed historical landscape genetics study at the population level for these island endemics would place their estimated divergence times more exactly, with calibration to recent glacial/interglacial cycles. A broad study of the effects of projected future climate change on Cactaceae in Baja California (Benavides et al., 2021) found that seven species of *Cochemiea* s.l. and four species of *Mammillaria* s.s. may undergo range expansion as a result of climate change impacts. These findings suggest that the Mammilloid clade in Baja California has perhaps been resilient to the repeated cooling and warming cycles that characterized the Pleistocene. On the other hand, Breslin et al. (2020) found that the island endemic *Cochemiea halei* is likely to lose 53% of its highly restricted suitable habitat by 2100. Further research is needed to understand the impacts of climate on the Mammilloid clade on different spatial and temporal scales.

Island endemism in the Cactaceae is high in Baja California, both on Gulf islands and islands in the Pacific Ocean off the Baja California coast, with an endemic taxon from *Cochemiea* s.l. on nearly every island in the region (Craig, 1945; Rebman and Roberts, 2012; Pilbeam, 2015). There is apparent dispersal both across the peninsula and along both coasts from north to south. The most recently emerging subclade in *Cochemiea* (containing *Cochemiea grahamii*) originated in the Cape at 5 Ma, followed by a rapid series of dispersals involving the Gulf Coast, the Pacific Coast, back to the Sonoran Desert, and again, back to the Baja California peninsula. This zig‐zag history over a short time may also account for the pattern of island endemism in *Cochemiea*, where the genus is represented by at least one endemic taxon on almost every island, with ancestors left on islands as stepping stones for frugivorous birds.

In summary, the biogeographical and divergence time analyses strongly support the effects of peninsular rifting as a contributing factor to speciation by vicariance, and do not show support in *Cochemiea* for mid‐peninsular seaways but do suggest the possible vicariance effect of an insular Cape region, and may show the influence of more recent glacial/interglacial cycles. These geological events driving a complex history of both vicariance and dispersal, combined with the heterogeneous climate and topography of the peninsula, and the apparent ability of these cacti to adapt rapidly to novel environments, create a picture of multiple historical abiotic drivers of diversification for taxa of *Cochemiea* s.l. and the regional *Mammillaria*.

### Biotic factors in dispersal and speciation

The most likely mid‐to‐long range disperser of *Cochemiea* is frugivorous birds. The genus produces large, red fruit, shown to be particularly attractive to birds (Bregman, 1988). The seeds are contained in sugary, mucilaginous pith, with seeds of *Cochemiea halei*, for example, observed to remain stuck to paper for more than five months, a pattern of adherence that may lend itself to long range dispersal (Breslin, personal observations). However, even if fruit is ingested, studies have shown that passage through the digestive tract of birds (or mammals), known as endozoochory, has no negative effect on germination, and, in fact, removes inhibitors and can lead to higher rates of germination (Fleming and Sosa, 1994; Rojas‐Arechiga and Vazquez‐Yanes, 1999). Although the viability period of *Cochemiea* seeds is not known, seed from some *Mammillaria* species has been shown to be viable for at least eight years, in habitat (Santini and Martorell, 2013). If long term seed viability is a characteristic of *Cochemiea*, this may help account for widespread dispersal across multiple habitats. Intrinsic barriers to hybridization appear to be present in *Mammillaria* and *Cochemiea* in habitat. No intergeneric hybrids between sympatric *Mammillaria* and *Cochemiea* have been observed, and no intermediate forms are known among the taxa in *Cochemiea*, although the sterile, vegetative characters of some taxa are very similar, which makes definitely identifying intermediate forms challenging in the absence of flowers (Rebman, San Diego Natural History Museum, personal communication, 2020). The lack of any observed hybrids or apparent reticulate evolution stands in contrast to certain other genera in the Cactaceae, for example, *Opuntia* (L.) Mill. (cf. Majure et al., 2012). In general, *Cochemiea* species are assumed to be obligate outcrossing taxa, although some species do self, or, occasionally, individuals are capable of selfing (Craig, 1945; Pilbeam, 2015). This combination of mostly obligate outcrossing with apparently strong barriers to hybridization may be another contributing factor to the high species diversity in the genus. Population‐level genetic studies would provide further insight into the mechanisms that facilitate the high rate of sympatry in Baja California *Cochemiea*, with 14 of the 26 taxa endemic to Baja California are known to co‐occur with at least one other taxon. Sympatric speciation may contribute yet another cause of high speciation, in addition to vicariance and adaptive radiation to geographically nearby but climatically distinct regions.

### Morphological traits in *Cochemiea* s.l.

One of the main morphological differences between *Cochemiea* s.l. and the Baja California and Sonoran Desert *Mammillaria* is the presence of hooked spines in *Cochemiea*. Hooked spines are not universally present in *Cochemiea*—*C. halei* always has straight spines as do *C. macdougallii* and *C. conoidea*, and a few of the other taxa in *Cochemiea* are reported to have either hooked or straight spines. Hooked spines are also not always absent in *Mammillaria*—*M. senilis* in this study, for example, has hooked spines (Craig, 1945). Several taxa shown to be deeply nested in *Mammillaria* by Butterworth and Wallace (2004) also have hooked spines. Hooked spines form as a result of layers of quickly lignifying cells having a differential rate of production, always higher on the adaxial surface (Mauseth, 2006, Schlegel, 2009). While the BiSSE analysis suggested that hooked spines are correlated with increased diversification, the HiSSE analysis indicated that that correlation was probably spurious, since the addition of hidden character states almost eliminated that correlation. However, it may be the case that the development of hooked spines is an important part of a suite of morphological traits that contributed to the evolutionary success of *Cochemiea*, although it is not known what advantage hooked spines confer (J. Mauseth, University of Texas, personal observation, 2019, and J. Rebman, San Diego Natural History Museum, personal observation, 2020). Researchers have speculated that hooked spines aid in dispersal and vegetative reproduction, through attachment to animals and deposition at a distance, or that the hooks function as additional defense against herbivory. Formal studies of these hypotheses have not been conducted for the Mammilloid clade. An additional, speculative hypothesis is that hooks condense a higher quantity of atmospheric moisture in fog zones that then drips to the soil surface, increasing available water (Anderson, 2001). Recent research (Kundanati et al., 2022) found that the straight spines of the cactus *Oreocereus trolli* Kupper, which occurs in the fog deserts of the Andes in Argentina and Bolivia, channel a significant volume of water that coalesces first as dew. Future research on hooked spines facilitating increased available water for the root zone would be particularly relevant for Baja California *Cochemiea*, as several areas of diversity along the Pacific coast into the central desert feature regular fogs (Webb and Turner, 2015), and the often‐foggy Pacific Coast of Baja California is shown in our biogeographical reconstruction to be an important ancestral area (Figure 2).

Hernández‐Hernández et al. (2014) found that the shift from generalist pollinated flowers to specialist pollination was one of the drivers of increased diversification in Cactaceae in general, in addition to increased aridification and reduced atmospheric CO_2_ in the Late Miocene. Further research into the distinct vegetative morphology, floral morphology and pollination syndromes of *Cochemiea* s.l. would shed more light on a possible relationship among species richness, endemism, and floral traits.

### Diversification rates within the Mammilloid clade

TurboMEDUSA has been shown to exaggerate diversification rates when only extant taxa are used in the analysis (cf. e.g., Louca and Pennell, 2020), so some caution should be used for interpreting our results. However, the BAMM analysis largely corroborates the high rates of speciation we found using TurboMEDUSA (Table 1). BAMM has been shown to be resilient to incomplete taxon sampling, and to provide reasonably strong inference, especially regarding relative rates of diversification among closely related clades (Sun et al., 2020). Regardless of possible inflation of absolute rates of diversification in both the TurboMEDUSA and BAMM analyses, the indicated major differences in relative rates remain informative.

The background diversification rate for the angiosperms in general is estimated to be between 0.077 and 0.089 new lineages per million years (Magallón and Sanderson, 2007), although more recent studies (e.g., Bell et al., 2010; Tank et al., 2015) have raised doubt about the usefulness or accuracy of a generalized background rate of diversification, due to evidence of repeated cycles of rapid radiation. Regardless of the agreed upon backdrop, both *Cochemiea* s.l. and *Mammillaria* s.s. display high absolute rates of diversification in this study. If the background rates from Magallón and Sanderson (2007) are used for comparison, for example, and mean diversification rates are taken from the BAMM analysis, *Cochemiea* conservatively has a diversification rate 14 times the background, and Baja California *Mammillaria*, 12 times the background rate (Table 1). Arakaki et al. (2011) estimated the diversification rate of the core Mammilloid clade at 0.225 lineage/Myr, and the core Cactaceae at 0.232 lineage/Myr. Hernández‐Hernández et al. (2014) arrived at pure birth estimates of 0.620 (stem) and 0.638 (crown) for the Mammilloid clade. Comparatively, *Cochemiea* has a diversification rate conservatively five times Arakaki's estimate for the Mammilloid clade and the core Cactaceae, and the regional *Mammillaria* of Baja California and the Sonoran/Sinaloan Gulf coast, about five times that of the Mammilloid clade as a whole. Our estimates of high relative rates of diversification for *Cochemiea* s.l. relative to the Mammilloid clade and the Mammilloid clade relative to Cactaceae and angiosperms as a whole are consistent with the current high species diversity and peninsular and island endemism observed in these lineages, in light of their recent dispersal to the area and recent divergence times.

## CONCLUSIONS

Detailed multiple and mutually supportive analyses reveal rapid, geospatially complex and multiple radiations of the Mammilloid clade, and the genera *Mammillaria* and *Cochemiea* of northwestern Mexico and the southwestern United States. Our findings contribute to a broader understanding some of the likely mechanistic drivers of angiosperm diversification at multiple spatial and temporal scales. *Cochemiea* is shown to have initially diversified in the Cape region of Baja California, perhaps speciating due to the vicariance event of peninsular rifting. The drivers of high rates of diversification and endemism are likely to be geological and climate heterogeneity at multiple spatial and temporal scales on the Baja California peninsula and in the surrounding region. The presence of species of *Cochemiea* s.l. and *Mammillaria* s.s. in every available habitat on the Baja California peninsula in particular, combined with their recent arrival to several of their current ranges, suggests a capacity for rapid adaptation to markedly novel abiotic conditions. Extensive sympatry between *Mammillaria* s.s. and *Cochemiea* s.l. and within *Cochemiea*, with no evidence of hybridization, indicates strong reproductive barriers intrinsic to these plants; this, combined with obligate outcrossing, has also likely driven high rates of speciation. The genus *Cochemiea*, in particular, displays high species richness, a high degree of peninsular endemism and rapid diversification. Future research regarding the other genera in Cactaceae with high speciation and endemism on the Baja California peninsula will place these results in a larger context, and contribute to our understanding of the evolution and biogeography of the Cactaceae as a whole.

## AUTHOR CONTRIBUTIONS

P.B.B.: study design, data analysis, manuscript preparation, and figure preparation; M.F.W.: manuscript preparation, revision, and editing; L.C.M.: manuscript preparation, revisions, editing, and conceptualization.

The data and analyses presented here were also included in a chapter of P.B.B.'s Ph.D. dissertation, and have been significantly revised.

## CONFLICT OF INTEREST

The authors claim no conflicts of interest.

## Data Availability

Raw sequence data used for phylogenetic analyses is publicly available at NCBI: https://www.ncbi.nlm.nih.gov/bioproject/PRJNA671701. Range coding for ancestral ranges, the ultrametric tree used for downstream analysis, and the RevBayes scripts used for biogeographical and character analyses are available at Figshare: https://doi.org/10.6084/m9.figshare.20422254.

## References

[ajb216048-bib-0001] Alfaro M. E. , F. Santini , C. Brock , H. Alamillo , A. Dornburg , D. L. Rabosky , G. Carnevale , and L. J. Harmon . 2009. Nine exceptional radiations plus high turnover explain species diversity in jawed vertebrates. Proceedings of the National Academy of Sciences, USA 106: 13410–13414. 10.1073/pnas.0811087106 PMC2715324 19633192

[ajb216048-bib-0002] Anderson, C. L. , K. Bremer , and E. M. Friis . 2005. Dating phylogenetically basal eudicots using *RbcL* sequences and multiple fossil reference points. American Journal of Botany 92: 1737–1748. 21646091 10.3732/ajb.92.10.1737

[ajb216048-bib-0003] Anderson, E. F. 2001. The cactus family. Timber Press, Portland, Oregon, USA.

[ajb216048-bib-0004] Applequist, W. L , and R. S. Wallace . 2001. Phylogeny of the portulacaceous cohort based on *ndhf* sequence data. Systematic Botany 26: 406–419.

[ajb216048-bib-0005] Arakaki, M. , P. Christin , R. Nyffeler , A. Lendel , U. Eggli , R. M. Ogburn , E. Spriggs , et al. 2011. Contemporaneous and recent radiations of the world's major succulent plant lineages. Proceedings of the National Academy of Sciences, USA 108: 8379–8384. 10.1073/pnas.1100628108 PMC3100969 21536881

[ajb216048-bib-0006] Bárcenas, R. T. , H. M. Hernandez , P. Hernández‐Ledesma , and L. M. Montoya Gómez . 2021. *Chichimecactus* (Cactoideae, Cactaceae), a new genus based on molecular characterisation of highly endangered *Strombocactus* species. Phytotaxa 512: 12–27.

[ajb216048-bib-0007] Bárcenas, R. T. , C. Yesson , and J. A. Hawkins . 2011. Molecular systematics of the Cactaceae. Cladistics 27: 1–20. 34875796 10.1111/j.1096-0031.2011.00350.x

[ajb216048-bib-0008] Barthlott, W. , K. Burstedde , J. L. Geffert , P. L. Ibisch , N. Korotkova , A. Michach , M. D. Rafiqpoor , et al. 2015. Biogeography and biodiversity of cacti. Schumannia 7: 1–110.

[ajb216048-bib-0009] Beaulieu, J. M. , and B. C. O'Meara . 2016. Detecting hidden diversification shifts in models of trait‐dependent speciation and extinction. Systematic Biology 65: 583–601. 27016728 10.1093/sysbio/syw022

[ajb216048-bib-0010] Bell, C. D. , D. E. Soltis , and P. S. Soltis . 2010. The age and diversification of the angiosperms re‐revisited. American Journal of Botany 97: 1296–1303. 21616882 10.3732/ajb.0900346

[ajb216048-bib-0011] Benavides, E. , A. Breceda , and J. D. Anadón . 2021. Winners and losers in the predicted impact of climate change on cacti species in Baja California. Plant Ecology 222: 29–44.

[ajb216048-bib-0012] Bennett, S. E. K. 2013. The role of lift obliquity in formation of the Gulf of California, Ph.D. dissertation, University of California, Davis, California, USA.

[ajb216048-bib-0014] Bregman, R. 1988. Forms of seed dispersal in Cactaceae. Acta Botanica Neerlandica 37: 395–402.

[ajb216048-bib-0015] Breslin, P. B. , M. F. Wojciechowski , and F. Albuquerque . 2020. Projected climate change threatens significant range contraction of *Cochemiea halei* (Cactaceae), an island endemic, serpentine‐adapted plant species at risk of extinction. Ecology and Evolution 10: 13211–13224. 33304531 10.1002/ece3.6914 PMC7713919

[ajb216048-bib-0016] Breslin, P. B. , M. F. Wojciechowski , and L. C. Majure . 2021. Molecular phylogeny of the Mammilloid clade (Cactaceae) resolves the monophyly of *Mammillaria* . Taxon 70: 308–323.

[ajb216048-bib-0017] Butterworth, C. A. , J. H. Cota‐Sanchez , and R. S. Wallace . 2002. Molecular systematics of tribe Cacteae (Cactaceae: Cactoideae): A phylogeny based on *rpl16* intron sequence variation. Systematic Botany 27: 257–270.

[ajb216048-bib-0018] Butterworth, C. A. , and R. S. Wallace . 2004. Phylogenetic studies of *Mammillaria* (Cactaceae)—insights from chloroplast sequence variation and hypothesis testing using the parametric bootstrap. American Journal of Botany 91: 1086–1098. 21653464 10.3732/ajb.91.7.1086

[ajb216048-bib-0107] Chaboureau, A. C. , P. Sepulchre Y. Donnadieu , and A. Franc . 2014. Tectonic driven climate change and the diversification of angiosperms. Proceedings of the National Academy of Sciences, USA 111: 14066–14070. 10.1073/pnas.1324002111 PMC4191762 25225405

[ajb216048-bib-0021] Cota‐Sánchez, J. H , and M. C. Bomfim‐Patrício . 2010. Seed morphology, polyploidy and the evolutionary history of the epiphytic cactus *Rhipsalis baccifera* (Cactaceae). Polibotánica 29: 107–129.

[ajb216048-bib-0022] Craig, R. T. 1945. The Mammillaria handbook. Abbey Garden Press, Carpinteria, California, USA.

[ajb216048-bib-0023] Crane, P. R. , E. M. Friis , and K. R. Pedersen . 1995. The origin and early diversification of angiosperms. Nature 374: 27–33.

[ajb216048-bib-0024] Crozier, B. S. 2005. Systematics of Cactaceae Juss.: Phylogeny, cpDNA evolution, and classification, with emphasis on the genus *Mammillaria* Haw. Ph.D. dissertation, University of Texas, Austin, Texas, USA.

[ajb216048-bib-0025] Darriba D. , G. L. Taboada , R. Doallo , and D. Posada . 2012. jModelTest 2: More models, new heuristics and parallel computing. Nature Methods 9: 772. 10.1038/nmeth.2109 PMC4594756 22847109

[ajb216048-bib-0026] Davies, T. J. , T. G. Barraclough , M. W. Chase , P. S. Soltis , D. E. Soltis , and V. Savolainen . 2004. Darwin's abominable mystery: Insights from a supertree of the angiosperms. Proceedings of the National Academy of Sciences, USA 101: 1904–1909. 10.1073/pnas.0308127100 PMC357025 14766971

[ajb216048-bib-0027] Dicht, R. F. , and A. D. Lüthy . 2005. Coryphantha: Cacti of Mexico and southern USA. Springer‐Verlag, Berlin, Germany.

[ajb216048-bib-0028] Dolby, G. A. , S. E. K. Bennett , R. J. Dorsey , M. F. Stokes , B. R. Riddle , A. Lira‐Noriega , and B. T. Wilder . 2022. Integrating Earth–life systems: A geogenomic approach. Trends in Ecology & Evolution 37: 371–384. 35123816 10.1016/j.tree.2021.12.004

[ajb216048-bib-0029] Dolby, G. A. , S. E. K. Bennett , A. Lira‐Noriega , B. T. Wilder , and A. Munguia‐Vega . 2015. Assessing the geological and climatic forcing of biodiversity and evolution surrounding the Gulf of California. Journal of the Southwest 57: 391–456.

[ajb216048-bib-0030] Doyle, J. J. , and J. L. Doyle . 1987. A rapid DNA isolation procedure for small quantities of fresh leaf tissue. Phytochemical Bulletin 19: 11–15.

[ajb216048-bib-0031] Drummond, A. J. , S. Y.W. Ho , M. J. Phillips , and A. Rambaut . 2006. Relaxed phylogenetics and dating with confidence. PLoS Biology 4: 699–710. 10.1371/journal.pbio.0040088 PMC1395354 16683862

[ajb216048-bib-0032] Drummond, A. J. , and A. Rambaut . 2007. BEAST: Bayesian evolutionary analysis by sampling trees. Evolutionary Biology 7: 214. 17996036 10.1186/1471-2148-7-214 PMC2247476

[ajb216048-bib-0033] Drummond A. J. , M. A. Suchard , D. Xie , and A. Rambaut . 2012. Bayesian phylogenetics with BEAUti and the BEAST 1.7. Molecular Biology and Evolution 29: 1969–1973. 22367748 10.1093/molbev/mss075 PMC3408070

[ajb216048-bib-0034] Drummond, C. S. , R. J. Eastwood , S. T. S. Miotto , and C. E. Hughes . 2012. Multiple continental radiations and correlates of diversification in *Lupinus* (Leguminosae): Testing for key innovation with incomplete taxon sampling. Systematic Biology 61: 443–460. 22228799 10.1093/sysbio/syr126 PMC3329764

[ajb216048-bib-0035] Edwards, E. J. , R. Nyffeler , and M. J. Donoghue . 2005. Basal cactus phylogeny: Implications of *Pereskia* (Cactaceae) paraphyly for the transition to the cactus life form. American Journal of Botany 92: 1177–1188. 21646140 10.3732/ajb.92.7.1177

[ajb216048-bib-0036] Fleming, T. H. , and V. J. Sosa . 1994. Effects of nectarivorous and frugivorous mammals on reproductive success of plants. Journal of Mammalogy 75: 845–851.

[ajb216048-bib-0037] Garcia Morales, L. J. , R. Gonzáles Gonzáles , J. G. Jiménez , and D. Iamonico . 2020. A new species of *Cochemiea* (Cactaceae, Cacteae) from Sinaloa, Mexico. Acta Botánica Mexicana 127: e1626.

[ajb216048-bib-0038] Gibson, A. C. , and P. S. Noble . 1986. The cactus primer. Harvard University Press, Cambridge, Massachusetts, USA.

[ajb216048-bib-0039] Guindon, S. , and O. Gascuel . 2003. A simple, fast and accurate method to estimate large phylogenies by maximum‐likelihood. Systematic Biology 52: 696–704. 14530136 10.1080/10635150390235520

[ajb216048-bib-0040] Helenes, J. , and A. L. Carreño . 1999. Neogene sedimentary evolution of Baja California in relation to regional tectonics. Journal of South American Earth Sciences 12: 589–605.

[ajb216048-bib-0041] Hernández, H. M. , and C. Gomez‐Hinostrosa . 2015. *Mapping the cacti of Mexico: Their geographical distribution based on referenced records*, Part II, *Mammillaria* . Succulent plant research, 7. Dh Books, Milborne Port, UK.

[ajb216048-bib-0042] Hernández‐Hernández, T. , J. W. Brown , B. O. Schlumpberger , L. E. Eguiarte , and S. Magallón . 2014. Beyond aridification: Multiple explanations for the elevated diversification of cacti in the New World Succulent Biome. New Phytologist 202: 1382–1397. 24611540 10.1111/nph.12752

[ajb216048-bib-0043] Hershkovitz, M. A , and E. A. Zimmer . 1997. On the evolutionary origins of the cacti. Taxon 46: 217–232.

[ajb216048-bib-0044] Höhna, S. , W. Freyman , and E. Goldberg . 2019. State‐dependent diversification with BiSSE and MuSSE: Inference using the binary/multiple state‐dependent speciation and extinction (BiSSE/MuSSE) branching process. RevBayes Tutorial. Website: https://revbayes.github.io/tutorials/sse/bisse.html. [accessed 3 June 2021].

[ajb216048-bib-0045] Höhna, S. , W. Freyman , A. M. Wright , and M. J. Landis . 2022. Discrete morphology: Ancestral state estimation and testing for irreversibility. RevBayes Tutorial. Website: https://revbayes.github.io/tutorials/morph/morph_more.html. [accessed 1 March 2022].

[ajb216048-bib-0046] Höhna, S. , M. J. Landis , T. A. Heath , B. Boussau , N. Lartillot , B. R. Moore , J. P. Huelsenbeck , and F. Ronquist . 2016. RevBayes: Bayesian phylogenetic inference using graphical models and an interactive model‐specification language. Systematic Biology 65: 726–736. 27235697 10.1093/sysbio/syw021 PMC4911942

[ajb216048-bib-0047] Hunt, D. R. 2016. CITES Cactaceae checklist, 3rd ed. Royal Botanic Gardens, Kew, UK.

[ajb216048-bib-0048] Katoh, M. , and M. Kuma . 2002. MAFFT: A novel method for rapid multiple sequence alignment based on fast Fourier transform. Nucleic Acids Research 30: 3059–3066. 12136088 10.1093/nar/gkf436 PMC135756

[ajb216048-bib-0049] Kundanati, L. , N. G. Di Novo , G. Greco , S. Siboni , C. D. Volpe , A. Bagolini , and N. M. Pugno . 2022. Multifunctional roles of hairs and spines in old man of the Andes cactus: Droplet distant coalescence and mechanical strength. Physics of Fluids 34: 012003.

[ajb216048-bib-0050] Landis, J. B. , D. E. Soltis , Z. Li , H. E. Marx , M. S. Barker , D. C. Tank , and P. S. Soltis . 2018. Impact of whole‐genome duplication events on diversification rates in angiosperms. American Journal of Botany 105: 348–363. 29719043 10.1002/ajb2.1060

[ajb216048-bib-0051] Landis, M. J. , W. A. Freyman , and B. G. Baldwin . 2018. Retracing the Hawaiian silversword radiation despite phylogenetic, biogeographic, and paleogeographic uncertainty. Evolution 72: 2343–2359. 30198108 10.1111/evo.13594

[ajb216048-bib-0052] Landis, M. J. , N. J. Matzke , B. R. Moore , and J. P. Huelsenbeck . 2013. Bayesian analysis of biogeography when the number of areas is large. Systematic Biology 62: 789–804. 23736102 10.1093/sysbio/syt040 PMC4064008

[ajb216048-bib-0053] Louca, S. , and M. W. Pennell . 2020. Extant timetrees are consistent with a myriad of diversification histories. Nature 580: 502–505. 32322065 10.1038/s41586-020-2176-1

[ajb216048-bib-0054] Maddison, W. P. , and D. R. Maddison . 2019. Mesquite: A modular system for evolutionary analysis, version 3.6. Website: http://www.mesquiteproject.org

[ajb216048-bib-0055] Maddison, W. P. , P. E. Midford , and S. P. Otto . 2007. Estimating a binary character's effect on speciation and extinction. Systematic Biology 56: 701–710. 17849325 10.1080/10635150701607033

[ajb216048-bib-0056] Magallón, S. , and A. Castillo . 2009. Angiosperm diversification through time. American Journal of Botany 96: 349–365. 21628193 10.3732/ajb.0800060

[ajb216048-bib-0057] Magallón, S. , S. Gómez‐Acevedo , L. L. Sánchez‐Reyes , and T. Hernández‐Hernandez . 2015. A metacalibrated time‐tree documents the early rise of flowering plant phylogenetic diversity. New Phytologist 207: 437–453. 25615647 10.1111/nph.13264

[ajb216048-bib-0058] Magallón, S. , and M. J. Sanderson . 2007. Absolute diversification rates in angiosperm clades. Evolution 55: 1762–1780. 10.1111/j.0014-3820.2001.tb00826.x 11681732

[ajb216048-bib-0060] Majure, L. C. , M. A. Baker , M. Cloud‐Hughes , A. Salywon , and K. M. Neubig . 2019. Phylogenomics in Cactaceae: A case study using the chollas sensu lato (Cylindropuntieae, Opuntioideae) reveals a common pattern out of the Chihuahuan and Sonoran deserts. American Journal of Botany 106: 1327–1345. 31545882 10.1002/ajb2.1364

[ajb216048-bib-0061] Majure, L. C. , R. Puente , M. P. Griffith , W. S. Judd , P. S. Soltis , and D. E. Soltis . 2012. Phylogeny of *Opuntia* s.s. (Cactaceae): Clade delineation, geographic origins, reticulate evolution. American Journal of Botany 99: 847–864. 22539520 10.3732/ajb.1100375

[ajb216048-bib-0062] Mauseth, J. D. 1990. Continental drift, climate and the evolution of cacti. Cactus and Succulent Journal 62: 302–308.

[ajb216048-bib-0063] Mauseth, J. D. 2006. Structure‐function relationships in highly modified shoots of Cactaceae. Annals of Botany 98: 901–926. 16820405 10.1093/aob/mcl133 PMC2803597

[ajb216048-bib-0064] Neubig, K. M. , W. M. Whitten , J. R. Abbott , S. Elliott , D. E. Soltis , and P. S. Soltis . 2014. Variables affecting DNA preservation in archival DNA specimens. *In* W. L. Applequist , and L. M. Campbell [eds.], DNA banking in the 21st century: Proceedings of the U.S. workshop on DNA banking, 81–136. William L. Brown Center, Missouri Botanical Garden. Saint Louis, Missouri, USA.

[ajb216048-bib-0065] Nobel, P. S. 2002. Cacti: Biology and uses. University of California Press, Berkeley, California, USA.

[ajb216048-bib-0066] Ocampo, G. , and J. T. Columbus . 2010. Molecular phylogenetics of suborder Cactineae (Caryophyllales), including insights into photosynthetic diversification and historical biogeography. American Journal of Botany 97: 1827–1847. 21616822 10.3732/ajb.1000227

[ajb216048-bib-0067] Olson, D. M. , E. Dinerstein , E. D. Wikramanayake , N. D. Burgess , G. V. N. Powell , E. C. Underwood , J. A. D'amico , et al. 2001. Terrestrial ecoregions of the world: A new map of life on Earth. Bioscience 51: 933–938.

[ajb216048-bib-0068] Paradis, E. , and K. Schliep . 2019. ape 5.0: An environment for modern phylogenetics and evolutionary analyses in R. Bioinformatics 35: 526–528. 30016406 10.1093/bioinformatics/bty633

[ajb216048-bib-0069] Pennell, M.W. , J.M. Eastman , G.J. Slater , J.W. Brown , J.C. Uyeda , R.G. FitzJohn , M.E. Alfaro , et al. 2014. geiger v2.0: an expanded suite of methods for fitting macroevolutionary models to phylogenetic trees. Bioinformatics 30(15): 2216–2218. 10.1093/bionformatics/btu181 24728855

[ajb216048-bib-0070] Pilbeam, J. 2015. *Cacti & succulents of Baja California*. British Cactus and Succulent Society, Essex, UK.

[ajb216048-bib-0106] R Core Team . 2021. R: A language and environment for statistical computing, version 4.0.5. R Foundation for Statistical Computing, Vianna, Austria. Website: https://www.R-project.org/.

[ajb216048-bib-0072] Rabosky, D. L. 2014. Automatic detection of key innovations, rate shifts, and diversity‐dependence on phylogenetic trees. PLoS One 9: 1–15. 10.1371/journal.pone.0089543 PMC3935878 24586858

[ajb216048-bib-0073] Rabosky D. L. , M. C. Grundler , C. J. Anderson , P. O. Title , J. J. Shi , J. W. Brown , H. Huang , and J. G. Larson . 2014. BAMMtools: An R package for the analysis of evolutionary dynamics on phylogenetic trees. Methods in Ecology and Evolution 5: 701–707.

[ajb216048-bib-0074] Rambaut A. , A. J. Drummond , D. Xie , G. Baele , and M. A. Suchard . 2018. Posterior summarisation in Bayesian phylogenetics using Tracer 1.7. Systematic Biology 67: 901–904. 29718447 10.1093/sysbio/syy032 PMC6101584

[ajb216048-bib-0075] Rebman, J. P. , and N. C. Roberts . 2012. Baja California plant field guide, 3^rd^ ed. Sunbelt Publications, San Diego, California, USA.

[ajb216048-bib-0076] Ree, R. H. , B. R. Moore , C. O. Webb , and M. J. Donoghue . 2005. A likelihood framework for inferring geographic range on phylogenetic trees. Evolution 59: 2299–2311. 16396171

[ajb216048-bib-0077] Ree, R. H. , and S. A. Smith . 2008. Maximum likelihood inference of geographic range evolution by dispersal, local extinction, and cladogenesis. Systematic Biology 57: 4–14. 18253896 10.1080/10635150701883881

[ajb216048-bib-0078] Riddle, B. R. , D. J. Hafner , L. F. Alexander , and J. R. Jaeger . 2000. Cryptic vicariance in the historical assembly of a Baja California peninsular desert biota. Proceedings of the National Academy of Sciences, USA 97: 1–6. 10.1073/pnas.250413397 PMC18937 11095731

[ajb216048-bib-0079] Riemann, H. , and E. Ezcurra . 2007. Endemic regions of the vascular flora of the peninsula of Baja California, Mexico. Journal of Vegetation Science 18: 327–336.

[ajb216048-bib-0080] Ripma L. A. , M. G. Simpson , and K. Hasenstab‐Lehman . 2014. Geneious! Simplified genome skimming methods for phylogenetic systematic studies: A case study in *Oreocarya* (Boraginaceae). Applications in Plant Sciences 2: 1–12. 10.3732/apps.1400062 PMC4259456 25506521

[ajb216048-bib-0081] Rojas‐Arechiga, M. , and C. Vazquez‐Yanes . 1999. Cactus seed germination: A review. Journal of Arid Environments 44: 85–104.

[ajb216048-bib-0082] Sánchez, D. , B. Vázquez‐Benítez , M. Vázquez‐Sánchez , D. Aquino , and S. Arias . 2022. Phylogenetic relationships in *Coryphantha* and implications on *Pelecyphora* and *Escobaria* (Cacteae, Cactoideae, Cactaceae). Phytokeys 188: 115–165. 35106054 10.3897/phytokeys.188.75739 PMC8799629

[ajb216048-bib-0083] Sanmartín, I. , P. Van Der Mark , and F. Ronquist . 2008. Inferring dispersal: A Bayesian approach to phylogeny‐based island biogeography, with special reference to the Canary Islands. Journal of Biogeography 35: 428–449.

[ajb216048-bib-0084] Santini, B. A. , and C. Martorell . 2013. Does retained‐seed priming drive the evolution of serotiny in drylands? An assessment using the cactus *Mammillaria hernandezii* . American Journal of Botany 100: 365–373. 23345416 10.3732/ajb.1200106

[ajb216048-bib-0085] Schlegel, U. 2009. The composite structure of cactus spines. Bradleya 27: 129–138.

[ajb216048-bib-0086] Serrano‐Serrano, M. L. , J. Rolland , J. L. Clark , N. Salamin , and M. Perret . 2017. Hummingbird pollination and the diversification of angiosperms: An old and successful association in Gesneriaceae. Proceedings of the Royal Society, B, Biological Sciences 284: 20162816. 10.1098/rspb.2016.2816 PMC5394660 28381621

[ajb216048-bib-0087] Smith, S. A. , J. W. Brown , Y. Yang , R. Bruenn , C. P. Drummond , S. F. Brockington , J. F. Walker et al. 2018. Disparity, diversity, and duplications in the Caryophyllales. New Phytologist 217: 836–854. 28892163 10.1111/nph.14772

[ajb216048-bib-0088] Soltis, P. S. , and D. E. Soltis . 2004. The origin and diversification of angiosperms. American Journal of Botany 91: 1614–1626. 21652312 10.3732/ajb.91.10.1614

[ajb216048-bib-0089] Straub, S. C. K. , M. Parks , K. Weitemier , M. Fishbein , R. C. Cronn , and A. Liston . 2012. Navigating the tip of the genomic iceberg: Next‐generation sequencing for plant systematics. American Journal of Botany 99: 349–364. 22174336 10.3732/ajb.1100335

[ajb216048-bib-0090] Sun, M. , R. A. Folk , M. A. Gitzendanner , P. S. Soltis , Z. Chen , D. E. Soltis , and R. P. Guralnick . 2020. Estimating rates and patterns of diversification with incomplete sampling: A case study in the rosids. American Journal of Botany 107: 895–909. 32519354 10.1002/ajb2.1479 PMC7384126

[ajb216048-bib-0091] Swofford, D. L. 2002. PAUP*. Phylogenetic analysis using parsimony (*and other methods), version 4a, build 165. Sinauer Associates, Sunderland, Massachusetts, USA.

[ajb216048-bib-0092] Tank, D. C. , J. M. Eastman , M. W. Pennell , P. S. Soltis , D. E. Soltis , C. E. Hinchliff , J. W. Brown , et al. 2015. Nested radiations and the pulse of angiosperm diversification: Increased diversification rates often follow whole genome duplications. New Phytologist 207: 454–467. 26053261 10.1111/nph.13491

[ajb216048-bib-0093] Tribble, C. M. , W. A. Freyman , M. J. Landis , J. Y. Lim , J. Barido‐Sotani , B. Tore Kopperud , S. Höhna , and M. R. May . 2021. RevGadgets: An R package for visualizing Bayesian phylogenetic analyses from RevBayes. Methods in Ecology and Evolution 13: 314–323.

[ajb216048-bib-0094] Upton, D. E. , and R. W. Murphy . 1997. Phylogeny of the side‐blotched lizards (Phrynosomatidae:Uta) based on MtDNA sequences: Support for a midpeninsular seaway in Baja California. Molecular Phylogenetics and Evolution 8: 104–113. 9242598 10.1006/mpev.1996.0392

[ajb216048-bib-0095] Vamosi, J. C. , and S. Vamosi . 2011. Factors influencing diversification in angiosperms: At the crossroads of intrinsic and extrinsic traits. American Journal of Botany 98: 460–471. 21613139 10.3732/ajb.1000311

[ajb216048-bib-0096] van der Niet, T. and S. D. Johnson . 2012. Phylogenetic evidence for pollinator‐driven diversification of angiosperms. Trends in Ecology and Evolution 27: 353–361. 22445687 10.1016/j.tree.2012.02.002

[ajb216048-bib-0097] Vázquez‐Sánchez, M. , D. Sánchez , T. Terazas , A. De La Rosa‐Tilapa , and S. Arias . 2019. Polyphyly of the iconic cactus genus *Turbinicarpus* (Cactaceae) and its generic circumscription. Botanical Journal of the Linnean Society, Linnean Society of London 190: 405–420.

[ajb216048-bib-0098] Vázquez‐Sánchez, M. , T. Terrazas , S. Arias , and H. Ochoterena . 2013. Molecular phylogeny, origin, and taxonomic implications of tribe Cacteae (Cactaceae). Systematics and Biodiversity 11: 103–116.

[ajb216048-bib-0099] Wallace, R. S. 1995. Molecular systematic study of the Cactaceae: Using chloroplast DNA variation to elucidate cactus phylogeny. Bradleya 1995: 1–12.

[ajb216048-bib-0100] Webb, R. H. , and R. M. Turner . 2015. Biodiversity of cacti and other succulent plants in Baja California, Mexico. Cactus and Succulent Journal 87: 206–216.

[ajb216048-bib-0101] Yao, G. , J.‐J. Jin , H.‐T. Li , J.‐B. Yang , V. S. Mandala , M. Croley , R. Mostow , et al. 2019. Plastid phylogenomic insights into the evolution of Caryophyllales. Molecular Phylogenetics and Evolution 134: 74–86. 30735725 10.1016/j.ympev.2018.12.023

[ajb216048-bib-0102] Yu, Y. , A. J. Harris , C. Blair , and X. He . 2020. RASP 4: Ancestral state reconstruction tool for multiple genes and characters. Molecular Biology and Evolution 37: 604–606. 31670774 10.1093/molbev/msz257

[ajb216048-bib-0103] Yu, Y. , A. J. Harris , and X. He . 2010. S‐DIVA (Statistical Dispersal‐Vicariance Analysis): A tool for inferring biogeographic histories. Molecular Phylogenetics and Evolution 56: 848–850. 20399277 10.1016/j.ympev.2010.04.011

